# Molecular biomarkers associated with respiratory insufficiency in amyotrophic lateral sclerosis

**DOI:** 10.1080/07853890.2021.1894009

**Published:** 2021-09-28

**Authors:** Ana Catarina Pronto-Laborinho, Catarina S. Lopes, Nuno C. Santos, Filomena A. Carvalho, Mamede de Carvalho

**Affiliations:** a Instituto de Medicina Molecular, Faculdade de Medicina, Universidade de Lisboa, Lisboa, Portugal; b Departmento de Neurociências e Saúde Mental, Hospital de Santa Maria-Centro Hospitalar Lisboa Norte, Lisboa, Portugal

## Abstract

Amyotrophic lateral sclerosis (ALS) is a devastating and fatal neurodegenerative disorder.Death typically occurs within 3–5 years after disease onset. The main cause of death in ALS is respiratory failure (RF). No effective treatment is available and no molecular biomarker related to respiratory outcome and to early ventilatory dysfunction was described so far. The club-cell protein (CC-16) is a biomarker associated with respiratory distress and lung inflammation.

The aim of this work is to test if CC-16 and IL-6 could be new biomarkers of ALS for early signs of respiratory insufficiency and disease progression. Additionally, we intend to study morphological and viscoelastic changes of the erythrocytes’ membrane associating them with ALS patients’ clinical profile.

Eighty-one ALS patients and 30 matched controls were included. Functional capacity and respiratory function (forced vital capacity) were evaluated. CC-16 and IL-6 were quantified by ELISA and multiplex technology, respectively. Morphological and viscoelastic properties of the erythrocytes were analysed by Atomic Force Microscopy (AFM).

CC-16 levels were significantly raised in ALS patients. In 17% of them, CC-16 level was above the upper cut-off value. On these patients, the risk of non-invasive ventilation was greater in the following 6 months and they tend to have higher mortality in the following 30 months. IL-6 values were not different in ALS population as compared with controls.

ALS patients have higher erythrocyte maximum height, area and volume, decreased erythrocyte membrane roughness and increased membrane stiffness than the control group. These results indicates abnormal erythrocyte structure and possible changes on membrane lipid composition on ALS patients.

We propose that increased CC-16 levels could be a marker of lung inflammatory response, associated with ventilatory insufficiency and related to impending respiratory failure, which are not fully predicted by conventional respiratory tests. Moreover, abnormalities in erythrocyte morphology may enhance the risk of tissue hypoxia.

Amyotrophic lateral sclerosis (ALS) is a devastating and fatal neurodegenerative disorder. Death typically occurs within 3–5 years after disease onset. Respiratory failure (RF) is the main cause of death. No effective treatment and no molecular biomarker related to respiratory outcome and to early ventilatory dysfunction are available. The club-cell protein (CC-16) is a biomarker associated with respiratory distress and lung inflammation.

The aim of this work is to test if CC-16 and IL-6 could be new biomarkers of ALS for early signs of respiratory insufficiency and disease progression. Additionally, we intend to study morphological and viscoelastic changes of the erythrocytes’ membrane associating them with ALS patients’ clinical profile.

Functional capacity and respiratory function (forced vital capacity) were evaluated in 81 ALS patients and 30 matched controls. CC-16 and IL-6 were quantified by ELISA and Multiplex-technology, respectively. Morphological and viscoelastic properties of the erythrocytes were analysed by Atomic Force Microscopy (AFM).

A significant negative correlation was found between [IL-6] and PhrAMPL, but not with ALSFRS-R or disease duration. CC-16 levels were significantly raised in ALS patients. In 17% of them, CC-16 level was above the upper cut-off value. On these patients, the risk of non-invasive ventilation was greater in the following 6 months and they tend to have higher mortality. ALS patients have higher erythrocyte maximum height, area, volume, decreased erythrocyte membrane roughness and increased membrane stiffness.

These results indicates that abnormal erythrocyte structure and possible changes on membrane lipid composition on ALS patients. Our results show that IL-6 levels are not dependent on the duration or severity of the disease, however, IL-6 may provide a marker of respiratory dysfunction in ALS. We propose that increased CC-16 levels could be a marker of lung inflammatory response, associated with ventilatory insufficiency and related to impending RF, which are not fully predicted by conventional respiratory tests. Moreover, abnormalities in erythrocyte morphology may enhance the risk of tissue hypoxia.

